# A Prospective Study of the Incidence and Characteristics of Post-Obstructive Diuresis Following Pyeloplasty for Unilateral Pelvi-Ureteric Junction Obstruction

**DOI:** 10.5152/tud.2026.25023

**Published:** 2026-03-13

**Authors:** Ashitosh D Pokharkar, Rohit Bhashkar Meshram, Charu Yadav, Amit Gupta, Partap S Yadav, Subhasis Roy Choudhury, Rajiv Chadha

**Affiliations:** Department of Pediatric Surgery, Lady Hardinge Medical College, Kalawati Saran Children’s Hospital, New Delhi, India

**Keywords:** Pelviureteric junction obstruction, polyuria, post-obstructive diuresis, pyeloplasty

## Abstract

**Objectives:**

To study the characteristics of urine production and post-obstructive diuresis (POD) following pyeloplasty for unilateral pelvi-ureteric junction obstruction (PUJO) in children and identify risk factors for the development of significant POD.

**Methods:**

A prospective observational study was conducted on pediatric patients undergoing pyeloplasty for unilateral PUJO. Post-operative urine output was measured from the affected kidney via nephrostomy catheter placed during surgery and from the opposite normal kidney via per-urethral catheter. Biochemical analysis of the urine sample was performed. Patients were divided into 2 groups: Group-1 with POD (total urine output (UO) >4 mL/kg/hour) (n = 19; 63.33%), and Group-2 (without POD) (n = 11; 36.66%). Preoperative imaging findings were analyzed in the 2 groups which may indicate risk for POD. Hydration was maintained in all patients with POD and remained stable with appropriate oral replacement.

**Results:**

Post-obstructive diuresis occurred in Group-1 patients within 48 hours after surgery and resolved by postoperative day 5. They had statistically significant lower median renal parenchymal thickness and higher median renal pelvis antero-posterior diameter (APD), differential ratio of kidney size and proportion of grade-4 hydronephrosis (HN) than Group-2 patients. Urine from the affected kidney had significantly higher mean UO, pH, fractional excretion of sodium (FeNa+), potassium (FeK+), magnesium (FeMg+) and lower specific gravity, creatinine clearance, (phosphorus) FePO_4_ than urine from the normal kidney.

**Conclusion:**

Post-obstructive diuresis occurring in children with pyeloplasty for unilateral PUJO is usually well tolerated by maintaining hydration. Patients with larger kidneys, more severe HN, higher pelvis APD, and parenchymal thinning are at a significantly higher risk.

**Main Points**

- Post-obstructive diuresis (POD) from the affected kidney is not uncommon in the initial postoperative days following pyeloplasty for unilateral PUJO.
- It is usually well tolerated if hydration is maintained with simple measures of replacing excess volume loss orally with oral rehydration solution and does not cause significant hemodynamic or metabolic derangement.
- Patients with larger kidneys and more severe hydronephrosis, higher renal pelvis antero-posterior diameter, and thinner renal parenchyma have a significantly higher risk of developing POD and merit close monitoring.
- The multiplicity of factors affecting renal function and outcome variables needs targeted and concerted research in the future with larger sample sizes and more precise definitions to understand the pathophysiology better.
- This would also help formulate a strategy for postoperative monitoring and management of these patients.
- Though this study has limited clinical relevance in patients with contralateral normal kidney, where diuresis often goes unnoticed, it has significant value for those with an unrecognized contralateral diseased kidney, who warrant close monitoring.
- This study provides foundational knowledge regarding the physiology of diuresis in humans with obstructive uropathy, a subject that has been rarely investigated.

## Introduction

Pelvi-ureteric junction obstruction (PUJO) is a common cause of hydronephrosis (HN) in children, and approximately one-third of antenatally detected cases require surgical intervention.^1^^,^^2^ Regardless of access, the Anderson–Hynes dismembered pyeloplasty technique is the most preferred surgical procedure and the gold standard against which all other interventions are compared.

Post-obstructive diuresis (POD) is a polyuric state in which large quantities of salt and water are eliminated after relief of urinary tract obstruction.^3^ Post-obstructive diuresis is a well-recognized phenomenon in bilateral ureteral obstruction but is considered relatively uncommon in unilateral PUJO due to compensation by the unaffected kidney.^4^^-^^6^ Post-obstructive diuresis is of 2 types: physiological and pathological. Physiologic POD is usually self-limiting, lasting for 24 hours or less, whereas pathologic POD lasts longer than 48 hours.^6^ Physiologic POD occurs due to excretion of accumulated solutes and free water from volume expansion that occurred during obstruction.^4^^,^^7^ Pathologic POD is characterized by prolonged and inappropriate handling of water and/or solutes and can be due to down regulation of sodium transport and aquaporin channels, poor responsiveness of the collecting ducts to vasopressin leading to nephrogenic diabetes insipidus, or altered regulation of atrial natriuretic peptide.^5^^,^^8^

In children with unilateral PUJO and a normal contralateral kidney, prolonged POD following relief of obstruction has been defined and described in case reports^9^^,^^10^ and also in some studies.^11^^-^^14^ This is important because although the POD following pyeloplasty is usually self-limiting and resolves without any complication, it can occasionally lead to significant dehydration with hypovolemia and electrolyte abnormalities necessitating correction to maintain homeostasis.^9^^,^^14^ Thus, pathologic POD may require close monitoring of vital signs, urine output (UO), serum electrolytes, and other metabolic parameters and consultation with nephrologists.

There are very few detailed studies on POD after unilateral pyeloplasty for patients with PUJO and a normal contralateral kidney. Apart from a prospective study by Li et al,^12^ the other 3 relatively large studies are retrospective in nature with limited data for analysis.^11^^,^^13^^,^^14^ Some earlier studies carried out to understand the abnormal pathophysiology and metabolic consequences of this condition have been performed on laboratory animals.^15^^-^^18^ As an external trans-anastomotic stent along with a nephrostomy tube (NT) was routinely placed following pyeloplasties at the center, POD from the affected kidney had been observed in several patients undergoing pyeloplasty for unilateral PUJO. Therefore, this prospective observational study in human pediatric patients was planned to comprehensively study the characteristics of urine production and POD following pyeloplasty for unilateral PUJO and attempt to identify the patient population that is at risk for the development of significant POD following surgical relief of obstruction.

## Material and Methods

After obtaining Institutional Ethical Committee clearance (No. LHMC/IEC/2019/37), this prospective observational study was conducted on children who underwent pyeloplasty for unilateral PUJO at a tertiary-care children’s hospital from December 2019 to November 2021. Initially, a study group of 50 patients managed over the 2-year period was decided as a convenient sample based on data from the preceding 10 years. But, owing to the COVID-19 pandemic with curtailment of elective surgeries, the study group was reduced to 30 patients.

The study group consisted of all children undergoing pyeloplasty for unilateral PUJO with a normal contralateral kidney during the study period. Patients having a solitary kidney with PUJO, unilateral PUJO associated with vesicoureteral reflux and/or vesicoureteral junction obstruction or other renal anomalies were excluded. A written informed parental consent was obtained before enrollment.

Clinical history was obtained with documentation of relevant clinical findings. Pre-operative demographic and imaging data were recorded. The parameters recorded on ultrasonography (USG) were: (i) antero-posterior diameter of the renal pelvis (APD), (ii) Society for Fetal Urology (SFU) grading of the hydronephrosis, (iii) renal parenchymal thickness in mm (PT) at mid-polar region, (iv) length and breadth/width of both kidneys, and (v) any findings regarding both ureters and the urinary bladder to rule out other associated anomalies. Two parameters calculated from the USG findings were: (i) differential size (cm), calculated as the difference of length between the affected and unaffected kidneys, and (ii) differential ratio, calculated by dividing the length of affected and unaffected kidneys.

Dynamic Renal Scanning (DRS) using Tc-99m L,L- Ethylene dicysteine or Tc-99m Diethyl Triamine Pentacetic Acid was used to assess the differential or split renal function (DRF/SRF) in percentage (%), the glomerular filtration rate (GFR) in mL/min/1.73m^2^, and the drainage, both pre- and postoperatively.

Open pyeloplasty was performed by the standard Anderson–Hynes dismembered technique with a trans-anastomotic stent (3-4.5 Fr umbilical catheter without side holes), an 8-10 Fr Foley catheter as NT (collected urine from affected kidney), and a perirenal drain. A per-urethral Foley catheter (PUC) of appropriate size was placed for bladder drainage (collected urine from contralateral normal kidney). The UO from the affected and the normal contralateral kidney was noted separately as reflected by the drainage into the NT and the PUC respectively. The drain was removed 24 hours after minimal or no drainage and the trans-anastomotic stent (ureteral stent) was removed on postoperative days 6-8 followed by clamping of the NT, which was removed 1 day later if the child remained asymptomatic. The urethral catheter was removed after the 5th postoperative day, following measurements of total output and various urinary parameters. 

Postoperative parameters recorded included any need for intravenous fluid administration beyond 12 hours after surgery (immediate postoperative period) or for nephrology consultation following POD. Urine output from the NT was considered as output from the affected kidney and urine collected by PUC drainage as urine from the contralateral kidney. Postoperative fluid given was half normal saline according to Holliday–Segar Method^19^ for at least 4 hours immediate post op and then discontinued after establishment of full feeds. Urine output from individual kidneys was recorded as mL/kg/hour every day (days 1-5) and the values added to get the total daily UO of the patient. The number of days required for normalization of UO was recorded.

Urinary specific gravity (SG), pH, sodium, potassium, magnesium, phosphates, and creatinine levels were documented separately from the NT and PUC on postoperative days 1, 3, and 5. The urinary SG, albumin, and pH were measured by UroColor 10 strips for urinalysis. The results were interpreted by a test results coding chart. Blood urea, serum creatinine, serum electrolytes, serum calcium, magnesium, and phosphate were measured on days 1, 3, and 5 postoperatively.

The following variables were calculated using standard formulas from the urinary and blood biochemistry for NT and PUC drainage separately: creatinine clearance and the fractional excretion of sodium (FeNa^+^), potassium (FeK^+^), phosphate (FePO_4_), and magnesium (Mg^+^) levels. All these values were calculated from samples of urine collected over 24 hours.

Postoperatively, the patients were observed closely for the development of POD. Post-obstructive diuresis was defined as total UO (NT and PUC output) of more than 4 mL/kg/hour.^20,21^ Accordingly, the patients were divided into 2 groups—Group-1 with POD and Group-2 without POD. Any occurrence of dyselectrolytemia was closely monitored in order to assess a need for nephrology consultation and intervention. The post-operative day on which the peri-renal drain, trans-anastomotic stent, NT, and PUC were removed was also recorded.

The primary outcome assessed was the incidence and characteristics of diuresis from the affected kidney following pyeloplasty for unilateral PUJO and, secondarily, the correlation of the findings with preoperative demographic data and nuclear scan and ultrasound (US) findings.

### Statistical Analysis

Statistical analysis was performed using SPSS statistical software version 17.0. Qualitative variables were analyzed using Chi-square test/Fisher exact test while quantitative variables were subjected to unpaired *T*-test and Mann–Whitney tests. Data was expressed as median (interquartile (IQR)) and range. *P*-value of less than .05 was taken as statistically significant.

## Results

Table 1 summarizes the demographic data and findings on preoperative imaging (USG and DRS). As many as 19 children (63.3%) were infants, there was a male predominance (n = 23; 76.7%) and the left kidney was more likely to be affected (n = 23; 76.7%).

Biochemical analysis of urine samples from the affected and the normal kidney during the postoperative period has been summarized in Table 2 as reflected by the drainage into the NT and the PUC respectively. No patient had significant albuminuria. Statistically significant observations comparing the affected kidney with the normal contralateral kidney were: higher mean UO, pH, FeNa+, FeK+, FeMg+ and lower SG, creatinine clearance, FePO4- (*P* < .05). The UO decreased gradually over the period of the study but the difference in values of all these parameters remained significant.

Table 3 represents the comparison between the 2 groups of patients: Group 1 with POD (63.33%; n = 19), and Group 2 without POD (36.66%; n = 11). Age was comparable in both groups. In Group 1 patients, the onset of POD occurred within 48 hours after surgery in all patients: 63.1% (n = 12) on day 1 & 36.9% (n = 7) on day 2. Diuresis lasted for more than 48 hours in 52.6% (n = 10) of patients and resolved by postoperative day 5 in all patients. Group 1 patients had a statistically significant lower median PT (*P *< .05) and higher median APD, differential ratio of the kidney size and proportion of SFU grade-4 HN (*P *< .05). However, there was no significant difference between the 2 groups with respect to preoperative SRF and GFR on DRS.

While comparing the 2 groups, the mean total UO was significantly higher in Group 1 than Group 2 on postoperative days 1 and 2 (*P *< .05), and while higher levels persisted in Group 1 patients on days 3-5, the difference was not statistically significant (Table 4). An important finding was that the difference in UO from NT and PUC was statistically significant in all patients (both Groups 1 and 2) on all 5 postoperative days (*P *< .05) Table 5. 

Seven patients (23.3%) developed pain and/or flank fullness on clamping the NT following removal of the trans-anastomotic stent. The NT was reopened and clamping restarted after a 3-day period. In these patients, the NT could be removed on postoperative days 10 to 12. No patient had any other significant perioperative or postoperative complication. As all patients remained hemodynamically stable with normal serum electrolyte levels, no intravenous fluid supplementation was required beyond the immediate postoperative period. Instead, to maintain adequate hydration, Oral Rehydration Solution (ORS) was administered at half the volume of calculated extra UO above 4 mL/kg/h to those with POD.

## Discussion

The etiology of POD following pyeloplasty for unilateral obstructive HN is likely multifactorial. It has been postulated that the damaged distal part of nephrons is unable to reabsorb water appropriately due to retained glomerular filtrate in the obstructed kidney in combination with the absence of normal urinary concentrating ability (UCA).^10^ Further, the POD following unilateral obstruction may be due to pressure atrophy of tissue near the collecting duct system, resulting in more tubular damage than glomerular damage.^22^ Tubular dysfunction at the level of proximal and distal convoluted tubules leads to decreased UCA, natriuresis, defect in urinary acidification, and deranged transport of other cations.^22^

Similar to the study protocol, Murer et al^11^ and Li et al^12^ measured the affected operated kidney’s output by NT drainage and the normal kidney’s output via a PUC. In other similar studies, the normal kidney’s output was measured by the voided output.^10^^,^^22^ In contrast, 2 other studies of POD following pyeloplasty for unilateral PUJO were retrospective with limited data regarding the differential UO.^13^^,^^14^ In the study, UO from the affected kidney was significantly higher than from the normal side on all 5 days of postoperative assessment with a declining trend over this period. Murer et al^11^ reported similar findings over a 5-day postoperative follow-up and Li et al^12^ over a 2-day follow-up. In a report of 3 newborns operated for unilateral PUJO, the affected kidney’s UO was >4 or >5 times that of the contralateral normal kidney.^9^ Thus, impaired UCA in the affected kidney after relief of obstruction appears to be a consistent finding.

The findings on blood and urinary biochemical analysis are mostly similar to those reported in related studies. Urine from the affected kidney had significantly lower SG as compared to that from the normal kidney, and similar findings have been reported earlier.^9^^,^^11^^,^^12^^,^^22^^,^^23^ Better et al^23^ reported urinary osmolality of 265 mOsm/kg water from the nephrostomy against 766 mOsm/kg water from the normal kidney following relief of complete urinary obstruction in a 24-year-old woman. These findings have been attributed to downregulation of AQP1 channels, resulting in impaired UCA of the affected kidneys with production of dilute urine having decreased SG.^24^ Selective downregulation of AQP2 in the post-obstructed kidney leads to increased excretion of hypotonic urine, and focal increase in PGE2 synthesis may be involved with AQP2 downregulation.^11^ Local increase in TGF-beta 1 level in the post-obstructed kidney may result in impairment in renal AQP1, which in turn can have a diuretic effect causing decreased urinary SG with reduced urinary exosomal AQP1 excretion.^12^

The significantly higher pH of the urine produced by the affected kidney in the study as well as in another report^22^ can be attributed to a defect in urinary acidification, and it has been demonstrated that the H^+^-ATPase down-regulation observed in unilateral ureteral obstruction is mediated by an increase in inducible Nitric oxide synthase, which itself appears to be regulated by Angiotensin 2.^25^

Significantly increased FeNa^+^ excretion in urine from the affected kidney may be explained by decreased sodium transport with major defects in renal tubular Na^+^ reabsorption localized to the distal segment of the nephron.^24^ A significant downregulation in Na^+^ transporters occurs within the nephron 24 hours after the onset of unilateral ureteral obstruction and occurs in the obstructed and unobstructed kidneys.^26^^-^^28^ The increase in excretion of sodium following relief of obstruction is believed to be due to inhibition of NaCl absorption by PGE2 in the thick ascending limb of the loop of Henle and vasopressin-induced increased permeability of water in the collecting ducts.^29^^,^^30^ As also reported earlier,^23^ the FeMg+ was significantly higher on the affected side than the unaffected side. Excessive loss of magnesium has been linked to excessive natriuresis following relief of obstruction in kidneys as calcium and magnesium resorption goes hand in hand with sodium resorption.^31^^,^^32^

The FeK^+^ excretion was significantly higher on the affected side on all postoperative days, and similar findings have been reported earlier.^33^ This is in contrast to the observations in experimental studies on rats^16^^,^^17^ and by a study on dogs^18^ which reported decreased potassium secretion in proportion to GFR after the release of a 24-hour period of unilateral ureteral obstruction. These contradictory findings are difficult to explain and merit further study.

In contrast to the increased fractional excretion of cations, urinary FePO4^-^ was significantly lower in the affected kidney as compared to the normal kidney over the study period, and this peculiar feature of unilateral obstruction, in contrast to bilateral obstruction, has been reported earlier.^22^^,^^34^ It has been stated that in humans, phosphate excretion from the post-obstructed kidney is consistently and markedly on the lower side after the release of unilateral ureteral obstruction, and this reduced excretion is out of proportion to the reduction in the filtered load.^34^

Creatinine clearance from the affected kidney improved substantially over 5 days following relief of obstruction, and similar findings have been reported earlier.^9^^,^^23^ In addition, creatinine clearance from the normal kidney decreased gradually over 3 days and remained static thereafter. A possible explanation is that the creatinine clearance of the normal kidney must have increased owing to obstruction in the diseased kidney to compensate for its malfunctioning, and following relief of obstruction decreased gradually as creatinine clearance of the affected kidney improved.

The incidence of significant POD (63%) in the study is quite higher than that reported in other similar, though retrospective, studies.^13^^,^^14^ Roth et al^14^ reported POD in 7/396 patients (1.8%) undergoing pyeloplasty for unilateral PUJO, while Bermeo et al^13^ found the incidence to be 30% (27/88 patients). A likely reason is that the definition for POD (> 4 mL/kg/hr), i.e. >200% of the expected normal UO for all ages, would tend to include more patients in the POD group as compared with the other studies where POD was defined as UO more than 300% of the expected normal UO according to age-defined criteria^14^ or as UO of >5 mL/kg/hr for 2 consecutive hours.^13^ The study also confirmed the findings reported earlier that patients with larger affected kidneys, higher SFU grade, and marked parenchymal thinning were more likely to have POD after relief of unilateral PUJO.^14^

Post-obstructive diuresis had resolved by postoperative day 5 in all Group 1 patients, suggesting that it is usually self-limiting. Significantly, patients not categorized as having POD (Group 2) also had NT urine output significantly higher than that from the normal kidney, although to a lesser degree. This suggests that some degree of diuresis is invariable following relief of obstruction, but the severity is variable. As both Groups 1 and 2 patients remained hemodynamically stable without significant metabolic or electrolyte changes throughout the postoperative study period, additional intravenous fluid infusion was not necessary to combat the extra losses. The administration of ORS can be considered as a precautionary measure sufficient to maintain hydration. In contrast, Roth et al^14^ reported that although the median time to resolution of postoperative changes was 3 days, 4/7 (57%) children with POD had metabolic and serum electrolyte abnormalities (hyponatremia = 2; hypophosphatemia = 1), and 1 patient had acidosis and lethargy. These patients needed nephrology consultation with additional intravenous fluids. Similarly, in another report, 3 newborns with POD following pyeloplasty for unilateral PUJO all needed intravenous fluid supplementation for marked diuresis for 3-9 days postoperatively.^9^

One of the lacunae of the study is that the sample size was relatively small. In addition, the definition of POD may have been too broad, accounting for the higher incidence of POD as compared to other studies.^13^^,^^14^ An inherent limitation in the study and in other similar studies^13^^,^^14^ is the assumption that all PUC urine was from the normal kidney. Although postoperative anastomotic edema and the presence of a trans-anastomotic stent would effectively minimize drainage of urine from the affected kidney into the urinary bladder in the initial postoperative days, the possibility of such occurrence to some degree cannot be ruled out. Measurement of more sophisticated urinary biomarkers, as in some earlier reports^11^^,^^12^ would also have provided more data for analysis and correlation.

### Artificial Intelligence Usage Statement:

The authors declared that no Artificial Intelligence Tool was used in the preparation of the manuscript.

## Figures and Tables

**Table 1.** Distribution of Patients According to Baseline Variables

Age (months)	3-6	9 (30%)
	6-12	10 (33.3%)
	12-36	8 (26.7%)
	>36-72	3 (10%)
Gender	Male	23 (76.7%)
	Female	7 (23.3%)
Weight (kg)	Median (IQR)	8.75 (IQR-4.12)
Laterality	Left	23 (76.7%)
	Right	7 (23.3%)
Preoperative Ultrasonography (USG)	Parenchymal thickness (mm)	3 (IQR-2)
	Differential size (mm)	2.35 (IQR-2.08)
	Differential ratio	1.5 (IQR-0.4)
	APD (mm)	33 (IQR-20)
	SFU Grade 3 Grade 4	17 (56.7%) 13 (43.3%)
Dynamic Renal scan (DRS)	Drainage Affected side Unaffected side	Obstructive (rising or plateau) Normal
	Differential function (%) Affected side Unaffected side	31.5 (IQR-18) 69.5 (IQR-16.75)
	GFR (ml/min/1.73m^2^) Affected side Unaffected side	26 (IQR-21) 57.5 (IQR-17.5)

APD, antero-posterior diameter; IQR, interquartile range; GFR, Glomerular Filtration Rate; SFU, Society for Fetal Urology.

**Table 2.** Post-Operative Biochemicalvariables

Variable	**Day 1**			**Day 3**			**Day 5**		
	**Nephrostomy**	**PUC**	*P*	**Nephrostomy**	**PUC**	*P*	**Nephrostomy**	**PUC**	*P*
Urine output (mL/kg/hr)	3 ± 1.07	1.96 ± 1.03	<.001	2.2 ± 0.83	1.01 ± 0.54	<.001	1.24 ± 0.47	0.6 ± 0.17	<.001
Specific gravity	1.01 ± 0.003	1.06 ± 0.11	<.001	1.01 ± 0.004	1.05 ± 0.008	<.001	1.02 ± 0.035	1.05 ± 0.07	<.001
Creatinine clearance (mg/mL/min)	22.81 ± 8.9	64.9 ± 5.1	<.001	27.1 ± 9.8	47.48 ± 6.29	.005	29.1 ± 9.9	45.6 ± 12.8	<.001
Ph	6.9 ± 0.28	6.0 ± 0.46	<.001	6.93 ± 0.25	5.78 ± 0.41	<.001	6.62 ± 0.57	5.68 ± 0.53	<.001
FeNa+	3.26 ± 0.94	1.57 ± 0.41	<.001	1.89 ± 0.63	0.93 ± 0.26	<.001	1.7 ± 0.24	0.79 ± 0.15	<.001
FeK+	17.51 ± 6.43	11.5 ± 4.6	<.001	16.3 ± 5.7	9.96 ± 4.6	<.001	13.2 ± 4.8	8.4 ± 3.2	<.001
FePO4−	11.5 ± 4.58	19.9 ± 6.4	<.001	9.2 ± 2.99	16.25 ± 4.16	<.001	9.96 ± 3.8	19.99 ± 5.23	<.001
FeMg+	8.63 ± 2.39	3.48 ± 0.89	<.001	5.9 ± 1.5	2.72 ± 0.95	<.001	6.26 ± 1.42	2.52 ± 0.69	<.001

PUC, per-urethral Foley catheter.

**Table 3.** Comparison of Preoperative Parameters Between Groups 1 and 2

**Preoperative Parameters**		**Group 1 (n = 19)**	**Group 2 (n = 11)**	*P*
Age in months	Median (IQR)	10 (17.5)	11 (12)	.948
Gender (n)	Male	14	9	1.0
	Female	5	2	
Weight (kg)	Median (IQR)	9 (3.5)	8 (4.5)	.488
Affected side (n)	Right	3	4	0.37
	Left	16	7	
Parenchymal thickness (mm)	Median (IQR)	3 (1)	4 (3)	.008*
Differential size (cm)	Median (IQR)	2.24 (2.05)	2 (2.75)	.084
Differential ratio	Median (IQR)	1.5 (0.55)	1.4 (0.3)	.007*
APD (mm)	Median (IQR)	35 (17)	21 (6.5)	<.001*
SRF of affected side (%)	Median (IQR)	34 (15.5)	31 (19)	.749
SFU Grade	Grade 3	3	10	<.001*
	Grade 4	16	1	
GFR of the affected side (mL/min/1.73m^2^)	Median (IQR)	24 (21)	28 (19.5)	.914

APD, antero-posterior diameter; IQR, interquartile range; GFR, Glomerular Filtration Rate; SFU, Society for Fetal Urology.

**Table 4.** Comparison of Daily Total Urine Output Between the 2 Groups (n = 30)

**Total Urine Output**	**Group 1 (n = 19)**	**Group 2 (n = 11)**	*P*
Day 1	5.53 ± 1.02	3.3 ± 0.75	.002*
Day 2	4.1 ± 0.86	3.29 ± 0.53	.025*
Day 3	3.52 ± 0.81	2.48 ± 0.72	.088
Day 4	2.52 ± 0.68	2.27 ± 0.56	.475
Day 5	1.89 ± 0.52	1.65 ± 0.38	.142

**Table 5.** Comparison Between Urine Output of Nephrostomy and PUC in Group 1 and Group 2

**Urine Output (mL/kg/hour)**	**Group 1**			**Group 2**		
	**Nephrostomy**	**PUC**	*P*	**Nephrostomy**	**PUC**	*P*
Day 1	3.41 ± 1.72	2.23 ± 1.18	**.003**	2.29 ± 0.36	1.48 ± 0.40	**.001**
Day 2	2.73 ± 1.04	1.37 ± 0.49	**<.001**	2.02 ± 0.56	1.28 ± 0.33	**.002**
Day 3	2.52 ± 1.12	1.2 ± 0.63	**<.001**	1.64 ± 0.50	0.83 ± 0.30	**<.001**
Day 4	1.70 ± 0.63	0.80 ± 0.28	**<.001**	1.40 ± 0.48	0.80 ± 0.19	**.001**
Day 5	1.35 ± 0.49	0.60 ± 0.21	**<.001**	1.06 ± 0.39	0.60 ± 0.10	**<.001**

PUC, per-urethral Foley catheter.

## Data Availability

The data that support the findings of this study are available on request from the corresponding author.
